# Pregnancy outcomes in idiopathic inflammatory myopathies: a Portuguese multicentre study

**DOI:** 10.3389/fmed.2025.1724170

**Published:** 2025-12-19

**Authors:** Ana Rita Lopes, Ana Teodósio Chícharo, Raquel Campanilho-Marques, Catarina Abreu, Sara Dias Rodrigues, Inês Santos, Filipa Paramés, Sofia C. Barreira, Maria Pulido-Valente, Luísa Pinto, Ana Rita Cruz-Machado, Susana Capela

**Affiliations:** 1 Rheumatology Department, ULS Santa Maria, Lisbon Academic Medical Center, Lisbon, Portugal; 2 Faculdade de Medicina, Universidade de Lisboa, Lisbon, Portugal; 3 Rheumatology Department, ULS Algarve, Faro, Portugal; 4 Rheumatology Department, ULS Almada-Seixal, Lisbon, Portugal; 5 Rheumatology Department, ULS Lisboa Ocidental, Lisbon, Portugal; 6 Rheumatology Department, ULS Viseu Dão-Lafões, Viseu, Portugal; 7 Clinical Pathology Department, ULS Santa Maria, Lisbon Academic Medical Center, Lisbon, Portugal; 8 Obstetrics, Gynaecology, and Reproductive Medicine Department, ULS Santa Maria, Lisbon Academic Medical Center, Lisbon, Portugal

**Keywords:** adverse pregnancy outcomes, disease flare, idiopathic inflammatory myopathies, maternal health, preconception, pregnancy

## Abstract

**Introduction:**

Idiopathic inflammatory myopathies (IIM) are rare autoimmune diseases that may complicate pregnancy, but evidence remains scarce. Disease activity at conception and during gestation, together with autoantibody profiles, are considered major determinants of adverse pregnancy outcomes (APOs).

**Methods:**

We conducted a retrospective multicentre study of 12 pregnancies in 10 women with IIM followed at four Portuguese tertiary centers (2009–2025). Demographic, clinical, immunological, therapeutic, maternal, and perinatal data were collected from medical records. Disease activity was assessed by clinical features, laboratory data, modified skin Disease Activity Score, and Manual Muscle Testing-8.

**Results:**

Phenotypes included overlap myositis (*n* = 4), antisynthetase syndrome (*n* = 3), dermatomyositis (*n* = 3), immune-mediated necrotising myopathy (*n* = 1), and polymyositis (*n* = 1). Myositis-specific/associated autoantibodies were detected in 91.7% of the cases, most frequently anti-Ro52 and anti-Jo-1. Disease remission at conception was documented in nine pregnancies (75.0%). Overall, APOs occurred in six pregnancies (50.0%): four miscarriages, one stillbirth, and one fetal growth restriction (FGR) with a small-for-gestational-age neonate. Of the 12 pregnancies, seven resulted in live births. All APOs occurred in pregnancies with disease flares, none occurred when disease remained stable. Among pregnancies with known preconception status, APOs occurred both in pregnancies conceived under active disease and in some conceived during remission. The most severe outcomes (stillbirth, FGR) occurred in one mother with antisynthetase syndrome and markedly elevated anti-Jo-1 titres. No cases of preeclampsia, eclampsia, congenital anomalies, or neonatal lupus were observed.

**Discussion:**

In this cohort, APOs were frequent, particularly among pregnancies complicated by disease activity. Although remission at conception was common, it did not fully prevent flares or obstetric complications. Anti-Jo-1 positivity appeared to cluster among the more severe cases, suggesting potential clinical relevance for monitoring. Multidisciplinary care and preconception optimization remain essential. Larger multicentre registries will be crucial to improving understanding and management of pregnancy in IIM.

## Introduction

Idiopathic inflammatory myopathies (IIM), encompassing dermatomyositis (DM), immune-mediated necrotising myopathy (IMNM), inclusion body myositis (IBM), antisynthetase syndrome (ASyS), overlap myositis, and polymyositis (PM), are rare autoimmune disorders characterized by chronic muscle inflammation and frequent multi-organ involvement, with heterogeneous treatment responses and outcomes (1, 2). These conditions predominantly affect women beyond their reproductive years, but can also occur during childbearing age, raising important concerns regarding fertility, pregnancy safety, and maternal-fetal outcomes (3–5).

In recent years, advances in immunosuppressive therapies, multidisciplinary care, and disease monitoring have allowed an increasing number of women with IIM to consider pregnancy. However, available evidence remains limited. Studies have highlighted that disease activity at conception and throughout gestation, as well as exposure to potentially teratogenic therapies, are major determinants of adverse pregnancy outcomes (APOs) (6). Reported complications include flares and hypertensive disorders, with preeclampsia rates reported up to 20% and cesarean delivery exceeding 60% in some cohorts. Fetal complications are also common, with fetal loss rates between 30–50% and preterm birth up to 30% (3, 4, 7).

The aim of this study was to characterize maternal and neonatal outcomes in women with IIM, with emphasis on preconception planning, immunological features, and pregnancy-compatible immunosuppression. Given prior evidence, we hypothesized that pregnancies complicated by disease activity—either at conception or during gestation—would be more frequently associated with APOs.

## Methods

### Study design and population

We conducted a retrospective observational study involving pregnant women diagnosed with IIM, managed at four Portuguese tertiary care centers between 2009 and 2025, following a nationwide data call. Eligible patients were adult women (≥18 years) with a confirmed diagnosis of IIM, established through 2017 ACR/EULAR classification criteria (8). Pregnancies were included whether IIM was diagnosed before or during gestation. *De novo* diagnoses during pregnancy were included because pregnancy can trigger or unmask IIM, and their outcomes are relevant for real-world clinical practice. Only pregnancies with complete clinical records were included.

### Data collection and definitions

Data were extracted from medical records after obtaining institutional ethics approval (reference 151/24). Variables collected included demographic data, IIM subtype, disease duration, clinical and laboratory indicators of disease activity and treatment at conception, throughout pregnancy and postpartum period. Disease activity was assessed based on clinical features (including characteristic cutaneous signs, proximal muscle involvement and other organ involvement), application of clinical scores—for muscle strength, the Manual Muscle Testing of eight groups (MMT8, score range 0–150, with high scores indicating minimal disease) (9) and the modified Disease Activity Score (DAS, score range 0–5, with low scores indicating minimal disease) (10) as a measure for skin disease activity. Modified skin DAS includes the four skin components of a simplified version of the original DAS tool, which are Gottron's papules (one point), heliotrope rash (one point), vasculitis (one point) and erythema (two points). Laboratory markers included muscle enzymes, specifically creatine kinase (CK) and aldolase. Remission was defined as the absence of clinical signs and symptoms, normalization of inflammatory markers and muscle enzyme levels, and stable organ function. Flare was defined as the onset or worsening of IIM-related manifestations during pregnancy or within 6 weeks postpartum. Missing data were recorded as ‘not available'; no imputation was performed.

### Outcomes

Maternal outcomes included miscarriage (< 22 weeks), stillbirth (≥22 weeks), preeclampsia (new-onset hypertension—systolic ≥140 mmHg or diastolic ≥90 mmHg—after 20 weeks of gestation or postpartum, accompanied by proteinuria >300 mg/24 h or maternal organ dysfunction), and eclampsia (generalized seizures in the context of preeclampsia) (11, 12).

Perinatal outcomes included preterm birth (< 37 weeks), fetal growth restriction (FGR, defined as estimated fetal weight < 3rd percentile, or < 10th percentile with abnormal Doppler findings), small for gestational age (SGA, birth weight < 10th percentile), congenital anomalies (structural malformations identified at birth), and neonatal infections occurring within the first 28 days of life (13, 14). Given the small sample size, only descriptive analyses were performed.

## Results

### Baseline patient characteristics and preconception status

We retrospectively reviewed 12 pregnancies in 10 women with confirmed IIM. Phenotypes included overlap myositis (*n* = 4), ASyS (*n* = 3), DM (*n* = 3), IMNM (*n* = 1), and PM, *n* = 1. Among overlap phenotypes, combinations were systemic sclerosis-PM (*n* = 2), rheumatoid arthritis-PM (*n* = 1), and PM-juvenile idiopathic arthritis (JIA, *n* = 1), with myositis phenotype being the predominant clinical feature.

Maternal characteristics at conception included a median age of 34.0 [5.5] years and a median disease duration of 5.0 [3.5] years. Seven women (58.3%) were primigravidae.

Major organ involvement before conception was identified in five pregnancies (41.7%), predominantly interstitial lung disease (ILD), observed in four pregnancies (33.3%) across three women, and gastrointestinal involvement, reported in one pregnancy (8.3%) in one woman. The first patient, with ASyS, had two pregnancies and an NSIP (nonspecific interstitial pneumonia) pattern with areas of organizing pneumonia on high-resolution computed tomography (HRCT). Pulmonary function was preserved (FVC 90%, FEV1 87%, DLCO 80.6%), and she remained asymptomatic throughout both gestations. The second ASyS patient also had ILD, presenting with exertional dyspnoea and dry cough. Preconception pulmonary function tests showed a restrictive ventilatory defect (FVC 64% predicted, TLC 83%, FEV1 67%, FEV1/FVC 90%) and reduced diffusion capacity (DLCO 54%, DLCO/VA 94%), HRCT demonstrated an NSIP pattern. This pregnancy ended in a first-trimester miscarriage before reassessment. The third patient, with overlap polymyositis–systemic sclerosis, was asymptomatic from a respiratory standpoint. Baseline HRCT showed an NSIP pattern and pulmonary function was normal prior to her first pregnancy. After that gestation, reassessment revealed preserved FVC (97%), mildly reduced DLCO (69%). She conceived again shortly afterwards, and a subsequent HRCT demonstrated mild interval progression of the NSIP pattern, while pulmonary function remained clinically stable.

Gastrointestinal disease was identified in one patient with DM, who developed dysphagia requiring IVIG, without evidence of oesophageal dysmotility, reflux-related complications, or nutritional impact.

No cardiac involvement or malignancy was documented. No patient developed new or worsening cardiopulmonary symptoms requiring repeat echocardiography or spirometry during pregnancy. Women with pre-existing ILD underwent pulmonary function testing and HRCT prior to conception as part of routine IIM follow-up. The most frequent manifestations were muscular (12 cases), cutaneous (nine), articular (seven), and vasculopathic features (ten), the latter referring exclusively to Raynaud's phenomenon. No digital ulcers, ischaemic lesions, or other vascular complications were reported, and no cases of calcinosis were identified.

Serological findings showed universal antinuclear antibodies (ANA) positivity and myositis-specific or myositis-associated antibodies were detected in 11 cases (91.7%), with some patients testing positive for multiple autoantibodies. The most frequent were anti-SSA (anti-Ro52) (*n* = 8), anti-PM/Scl-75 (*n* = 3), anti-Jo-1 (*n* = 3), anti-Mi-2α/β (*n* = 1), anti-TIF-1γ (*n* = 1), and anti-SRP (*n* = 1). No patient exhibited antiphospholipid antibodies.

Two pregnancies (16.7%) corresponded to *de novo* diagnoses established during the first trimester (1 DM and 1 IMNM), while the remaining ten pregnancies (83.3%) occurred in women with a previous diagnosis of IIM. Preconception counseling was recorded in seven of the ten pregnancies (70.0%) with known IIM before conception, indicating that almost one-third lacked counseling despite follow-up in tertiary centers. Among these ten pregnancies, disease remission at conception was observed in nine pregnancies.

Clinical phenotypes, autoantibody profiles, immunosuppressive regimens, disease course and maternal and perinatal outcomes are summarized in Tables 1–3 and Supplementary Tables S1–S2.

**Table 1:** Maternal, disease-related and preconception characteristics of the pregnancies in idiopathic inflammatory myopathies.

**Pregnancy**	**IIM Subtype**	**Autoantibodies**	**Major organ involvement**	**Age at conception, years**	**Disease duration, years**	**Disease status at conception**
1	Overlap (SSc-PM)	anti-PM/Scl-75, anti-Ro60, anti-SSB	ILD	32	5	Remission
2	Overlap (SSc-PM)	anti-PM/Scl-75, anti-Ro60, anti-SSB	ILD	33	6	Remission
3	Overlap (RA-PM)	anti-Ro52, anti-SSB	None	36	22	Remission
4	Overlap (PM-JIA)	anti-PM/Scl-75	None	38	12	Remission
5	ASyS	anti-Jo-1, anti-Ro52	ILD	29	7	Active
6	ASyS	anti-Jo-1, anti-Ro52	ILD	37	3	Active
7	ASyS	anti-Jo-1, anti-Ro52	ILD	38	4	Active
8	DM	anti-TIF-1γ, anti-Ro52	GI	31	0	N/A
9	DM	anti-Ro52	None	33	2	Remission
10	DM	anti-Mi-2α/β	None	41	5	Remission
11	IMNM	anti-SRP, anti-Ro52, anti-Ro60	None	25	0	N/A
12	PM	anti-Ro52	None	35	5	Remission

IIM, idiopathic inflammatory myopathies; ASyS, antisynthetase syndrome; DM, dermatomyositis; PM, polymyositis; IMNM, immune-mediated necrotising myopathy; RA, rheumatoid arthritis; SSc, systemic sclerosis; JIA, juvenile idiopathic arthritis; ILD, interstitial lung disease; GI, gastrointestinal involvement; N/A, not applicable.

**Table 2:** Adverse pregnancy outcomes observed in pregnancies in idiopathic inflammatory myopathies.

**Pregnancy**	**IIM subtype**	**APO type**	**Gestational age, weeks**	**Flare during pregnancy**	**Trimester of flare**
2	Overlap (SSc-PM)	Miscarriage	6	CK elevation, myalgia, proximal weakness	1^st^ trimester
4	Overlap (PM-JIA)	Miscarriage	10	Periorbital erythema flare	1^st^ trimester
5	ASyS	Miscarriage	6	CK elevation flare + Teratogenic exposure (MMF/CYC)	1^st^ trimester
6	ASyS	Stillbirth	37.1	Arthritis, mechanic's hands	3^rd^ trimester
7	ASyS	FGR + SGA	33.7	Arthritis, mechanic's hands	3^rd^ trimester
10	DM	Miscarriage	8	CK elevation flare	1^st^ trimester

IIM, idiopathic inflammatory myopathy; ASyS, antisynthetase syndrome; DM, dermatomyositis; PM, polymyositis; SSc, systemic sclerosis; JIA, juvenile idiopathic arthritis; APO, adverse pregnancy outcome; FGR, fetal growth restriction; SGA, small for gestational age; CK, creatine kinase; ILD, interstitial lung disease; MMF, mycophenolate mofetil; CYC, cyclophosphamide.

**Table 3:** Summary of maternal and perinatal outcomes by disease phenotype in idiopathic inflammatory myopathies.

**Outcome**	**DM (3)**	**IMNM (1)**	**ASyS (3)**	**PM (1)**	**Overlap (4)**	**Total IIM (12)**
Live births, *n* (%)	2 (66.7)	1 (100.0)	1 (33.3)	1 (100.0)	2 (50.0)	7 (58.3)
Early miscarriages and stillbirth, *n* (%)	1 (33.3)	0	2 (66.7)	0	2 (50.0)	5 (33.3)
Preterm births, *n* (%)	0	0	1 (33.3)	0	0	1 (8.3)
Birth weight (grams), median [IQR]	2910.0 [10.0]	3925.0	1745.0	3060.0	3217.5 [697.5]	2920.0 [777.5]
FGR or SGA, *n* (%)	0	0	1(33.3)	0	0	1 (8.3)
Cesarean section, *n* (%)	2 (66.7)	1 (100.0)	1 (33.3)	0	1 (25.0)	5 (41.7)
Preeclampsia or eclampsia, *n* (%)	0	0	0	0	0	0
Disease flares during pregnancy, *n* (%)	2 (66.7)	1 (100.0)	3 (100.0)	0	2 (50.0)	8 (66.7)
Disease flares in postpartum period, *n* (%)	0	1 (100.0)	1	0	0	2 (16.7)
Occurrence of adverse pregnancy outcomes, *n* (%)	1 (33.3)	0	3 (100.0)	0	2 (50.0)	6 (50.0)

DM, dermatomyositis; IMNM, immune-mediated necrotising myopathy; ASyS, antisynthetase syndrome; PM, polymyositis; IIM, idiopathic inflammatory myopathies; FGR, fetal growth restriction; SGA, small-for-gestational-age; IQR, interquartile range.

### Disease flares and evolution

A total of eight flares (66.7%) occurred during pregnancy. Six were true flares in women with pre-existing IIM, including three whose disease had been well controlled at conception (two with overlap myositis and one with DM). The remaining two episodes represented new-onset IIM diagnosed during pregnancy: one case of IMNM, presenting with proximal muscle weakness and elevated CK, and one case of DM, presenting with characteristic cutaneous rash, dysphagia and polyarthritis. Overall, flares most commonly manifested with CK elevation, musculoskeletal involvement (arthralgia, arthritis, myalgia or proximal weakness), and cutaneous features consistent with IIM. In contrast, four women (33%)—one with DM, one with PM and two with overlap myositis—remained in remission from preconception throughout pregnancy.

Two postpartum flares occurred and were limited to musculoskeletal involvement: one ASyS patient experienced worsening arthritis, and the IMNM patient developed myalgia and proximal weakness, both required increased prednisolone dosage and optimization of immunosuppressive therapy.

### Immunosuppressive management

Immunosuppressive management before and during pregnancy reflected both long-term disease control and flare-directed adjustments. All treatment modifications during pregnancy were prompted by maternal disease activity rather than obstetric complications.

Preconception immunosuppressive exposure: among the 10 women with established IIM prior to conception, all had been treated to glucocorticoids (GC), with a median prednisolone-equivalent daily dose of 5.0 mg/day [IQR 4.4]. Antimalarials were used in 7 (70.0%) women, azathioprine in 5 (50.0%), and other agents including mycophenolate mofetil, cyclosporine, cyclophosphamide, rituximab, and tocilizumab; according to phenotype and severity. Combination therapy was prescribed in 6 pregnancies (60.0%; 2 overlap, 3 ASyS and 1 DM), while 4 (40.0%) received monotherapy (2 overlap, 1 DM and 1 PM). The two women with *de novo* IIM had no exposure to disease-modifying therapy at conception.

Immunosuppressive therapy during pregnancy: conventional disease-modifying antirheumatic drugs (DMARDs) were used in 11 pregnancies (91.7%): azathioprine in six (3 overlap, 2 DM, 1 PM) and hydroxychloroquine in eight (4 overlap, 2 DM, 2 ASyS). Hydroxychloroquine was used in in most anti-SSA/B-positive women as part of preconception optimization or ongoing therapy, and in one DM patient as baseline disease management despite anti-SSA/B negativity. Biologic DMARDs and intravenous immunoglobulin (IVIG) were reserved for patients with severe or refractory disease. Rituximab had been administered before conception in one woman with progressive ILD, while tocilizumab was continued in another patient for control of refractory inflammatory arthritis. IVIG was initiated during pregnancy in the woman with DM who developed dysphagia. One unplanned pregnancy occurred shortly after exposure to mycophenolate and cyclophosphamide, both discontinued upon confirmation of gestation. This resulted in an early pregnancy loss.

GC use and flare-directed adjustments: GC were used in all pregnancies, with prednisolone-equivalent dose exceeded 7.5 mg/day in five pregnancies (42%), including three ASyS, one DM, and one overlap case. The cumulative dose ranged from 227.5 to 10760 mg [median 1343 (718–1874) mg]. Dose escalation was required in five of the eight flares (1 ASyS, 2 overlap myositis, 1 DM, and 1 IMNM). Among the six true flares in women with pre-existing IIM, prednisolone adjustments were modest (maximum of 10 mg/day), and remained unchanged in the remaining three cases. Management of *de novo* disease was tailored to clinical severity and organ involvement: both women received azathioprine, DM received three low-dose pulses of methylprednisolone (250 mg), followed by oral prednisolone 1 mg/kg/day with gradual tapering during pregnancy and IVIG (2 g/kg) due to gastrointestinal involvement with dysphagia. The IMNM remained on 5 mg/day of prednisolone throughout gestation.

Other medication adjustments: several women were taking proton pump inhibitors or calcium-channel blockers prior to conception for dyspepsia or Raynaud's phenomenon, respectively. All these agents were discontinued once pregnancy was confirmed, as no gastrointestinal or vascular manifestations required ongoing pharmacological treatment.

### Pregnancy and neonatal outcomes

A total of 12 pregnancies occurred in ten women with IIM. Two women had more than one pregnancy (one with ASyS and one with overlap PM-SSc); outcomes differed between pregnancies in both cases, and each pregnancy was analyzed individually.

APOs occurred in 6 pregnancies (50.0%). No APOs occurred in the two *de novo* diagnoses (DM and IMNM), both of which resulted in uncomplicated pregnancies until the time of diagnosis and were managed promptly without obstetric complications. The six APOs included four first-trimester miscarriages (one in ASyS, two in overlap (PM-SSc and PM-JIA) and one in DM), one stillbirth (ASyS), and one case of FGR with a SGA neonate (ASyS).

Importantly, no APO occurred in the absence of a maternal disease flare.

Live births occurred in seven pregnancies (58.3%). Median gestational age at delivery was 39.0 [1.0] weeks, with one preterm birth (33.7 weeks) due to fetal distress in the setting of active ASyS. Median birthweight was 2920.0 [777.5] g. No structural congenital anomalies, neonatal lupus, or early neonatal infections were observed.

Obstetric management followed high-risk pregnancy protocols. Low-dose aspirin was prescribed in six pregnancies (50.0%), in line with standard practice for women with autoimmune disease. One woman with a history of placental vascular pathology received prophylactic LMWH. Routine obstetric Doppler surveillance was performed in all pregnancies according to high-risk protocols and two cases of Doppler abnormalities indicative of placental insufficiency were detected in a woman with ASyS. All anti-SSA/B-positive pregnancies underwent serial fetal echocardiography, with no conduction abnormalities detected.

Regarding the delivery mode, cesarean section occurred in five of the seven live births (71.4%), most commonly for obstetric indications: prior cesarean section (*n* = 1), cephalopelvic disproportion (*n* = 2), and non-reassuring fetal cardiotocography (*n* = 2). The single preterm delivery (33.7 weeks) resulted from an emergency cesarean section due to fetal distress in active ASyS.

## Discussion

In this multicentre series, pregnancies complicated by IIM frequently presented APOs, mainly among women with disease activity during gestation, with all APOs occurring exclusively in pregnancies with maternal disease flares, reflecting a pattern consistently described in previous reports. This aligns with previous studies showing that clinical or subclinical activity, including isolated CK elevation, tends to associate with higher rates of miscarriage, stillbirth, preterm birth and low birth weight (3–5, 7, 15–23). Although conception during remission was associated with better outcomes, APOs still occurred in a minority of pregnancies conceived in remission, consistent with the literature (3, 4, 7, 15, 18). Population-based data also suggest that subclinical autoimmunity may impair placentation even before clinical onset of IIM (21).

Two women in our series presented with new-onset disease during pregnancy, illustrating that pregnancy may unmask or accelerate IIM, as previously reported (19, 23), particularly in DM, where immune modulation of gestation may trigger autoimmunity, sometimes described as associated with anti-TIF1-γ antibodies (23). Both required pregnancy-compatible immunosuppression, one required methylprednisolone pulses followed by high-dose oral prednisolone, combined with IVIG for dysphagia—illustrating the need for early recognition, rapid multidisciplinary assessment, and timely escalation when organ-threatening features emerge.

Autoantibody profiling provided clinically relevant stratification. The most severe outcomes—a stillbirth and a growth-restricted neonate—occurred in a mother with ASyS and markedly elevated anti-Jo-1 titres. Previous studies have also linked anti-Jo-1 positivity with higher systemic inflammatory burden and worse prognosis in myositis (15, 24). Longitudinal data suggest that anti-Jo-1 titers correlate with disease activity, supporting their use as potential markers of disease activity (25). These findings underscore the importance of intensified monitoring in Jo-1-positive pregnancies, particularly when disease control is suboptimal. Given the small sample size, this observation should be considered hypothesis-generating rather than definitive.

Our therapeutic patterns reflected contemporary practice. Low-dose glucocorticoids were widely used, with escalation during flares, while hydroxychloroquine and azathioprine were the most frequent background agents and were not associated with congenital anomalies or neonatal complications. IVIG and rituximab were reserved for refractory disease, consistent with current evidence (3, 6, 26). In contrast, inadvertent early-pregnancy exposure to mycophenolate and cyclophosphamide led to miscarriage, reinforcing the need for preconception counseling, effective contraception during teratogen use, and early pregnancy testing in women of reproductive potential (6, 27). The use of LDA aligns with current recommendations for women with autoimmune disease at increased risk of preeclampsia and placental dysfunction (28). Variation in LDA use reflects changes in recommendations over time and individualized obstetric risk assessment rather than differences in IIM phenotype or antiphospholipid antibody status. LMWH was prescribed in one case with a previous intrauterine fetal death associated with massive perivillous fibrin deposition, aiming to prevent recurrence of placental vascular pathology. While evidence for these strategies in IIM is scarce, extrapolation from systemic lupus erythematosus and antiphospholipid syndrome cohorts supports their application in selected high-risk scenarios (27, 29, 30).

Our results broadly align with international experience. Asian studies reported nearly universal APOs under active disease (3, 7); Hungarian and Swedish cohorts confirmed high fetal loss, preterm birth and cesarean rates (4, 15, 16, 22); and U.S. population-based studies highlighted additional maternal morbidity, especially hypertensive disorders findings parallel prior reports of increased cesarean delivery in increased cesarean delivery in post-IIM pregnancies (20, 21). Across cohorts, the consistent message is that active IIM—overt or subclinical—is frequently observed in pregnancies with complications, reinforcing guideline recommendations that disease should be in remission before conception and remain tightly controlled during pregnancy (6, 27).

Strengths of this study include being the first Portuguese multicentre pregnancy in IIM, spanning 15 years and integrating standardized clinical, immunological, and obstetric data. The use of disease activity metrics (e.g., modified skin DAS, MMT8) and detailed immunological phenotyping enhances external validity and comparability with international cohorts. Limitations relate mainly to the retrospective design, incomplete standardization of assessment intervals and the intrinsic rarity of IIM, which limits cohort size. As a descriptive observational study, it was not designed for inferential statistical comparisons, and statistical power considerations are not applicable. The absence of universally validated definitions for remission and flare in IIM required a pragmatic, clinically driven approach. Subclinical disease activity may also have been underestimated due to heterogeneous timing of assessments across centers. assessments across centers.

In conclusion, pregnancy in IIM should ideally be planned during remission, under pregnancy-compatible therapy, with vigilant monitoring by a multidisciplinary team throughout gestation. In this cohort, APOs were observed in the context of disease activity, and more severe outcomes occurred in one patient with high anti-Jo-1 titres. Our findings reinforce the need for strict disease control before conception and close surveillance in pregnancies complicated by ASyS. These findings highlight the importance of early identification of new-onset presentations, timely flare management and intensified surveillance in pregnancies complicated by ASyS. Multidisciplinary management and systematic preconception counseling remain essential strategies to optimize outcomes in women with IIM. Larger multicentre registries will be crucial to refine evidence-based guidance for this population.

## Data Availability

The original contributions presented in the study are included in the article/Supplementary material, further inquiries can be directed to the corresponding author.

## References

[1] Lundberg IE Fujimoto M Vencovsky J Aggarwal R Holmqvist M Christopher-Stine L . Idiopathic inflammatory myopathies. Nat Rev Dis Primers. (2021) 7:86. doi: 10.1038/s41572-021-00321-x 34857798

[2] Wu Y Luo J Duan L. Pathogenic mechanisms of disease in idiopathic inflammatory myopathies: autoantibodies as clues. Front Immunol. (2024) 15:1439807. doi: 10.3389/fimmu.2024.1439807 39281689 PMC11392717

[3] Zhong Z Lin F Yang J Zhang F Zeng X You X. Pregnancy in polymyositis or dermatomyositis: retrospective results from a tertiary centre in China. Rheumatology. (2017) 56:1272–5. doi: 10.1093/rheumatology/kex070 28387857

[4] Váncsa A Ponyi A Constantin T Zeher M Dankó K. Pregnancy outcome in idiopathic inflammatory myopathy. Rheumatol Int. (2007) 27:435–9. doi: 10.1007/s00296-006-0239-8 17033833

[5] Munira S Christopher-Stine L. Pregnancy in myositis and scleroderma. Best Pract Res Clin Obstet Gynaecol. (2020) 64:59–67. doi: 10.1016/j.bpobgyn.2019.10.004 31928915

[6] Oldroyd AGS Lilleker JB Amin T Aragon O Bechman K Cuthbert V . British Society for Rheumatology guideline on management of paediatric, adolescent and adult patients with idiopathic inflammatory myopathy. Rheumatology. (2022) 61:1760–8. doi: 10.1093/rheumatology/keac115 35355064 PMC9398208

[7] Mino R Shimada H Wakiya R Nakashima S Miyagi T Sugihara K . Pregnancy course and outcomes of patients with polymyositis and dermatomyositis (PM/DM) managed in a single center. Medicine. (2023) 102:e33462. doi: 10.1097/MD.0000000000033462 37026900 PMC10082272

[8] Lundberg IE Bottai M Tjärnlund A. 2017 European League Against Rheumatism/American College of Rheumatology classification criteria for adult and juvenile idiopathic inflammatory myopathies and their major subgroups. Ann Rheum Dis. (2017) 76:1955–64. doi: 10.1136/annrheumdis-2017-212786 29079590 PMC5736307

[9] Rider LG Koziol D Giannini EH Jain MS Smith MR Whitney-Mahoney K . Validation of manual muscle testing and a subset of eight muscles for adult and juvenile idiopathic inflammatory myopathies. Arthritis Care Res. (2010) 62:465–72. doi: 10.1002/acr.20035 20391500 PMC2924143

[10] Lam CG Manlhiot C Pullenayegum EM Feldman BM. Efficacy of intravenous Ig therapy in juvenile dermatomyositis. Ann Rheum Dis. (2011) 70:2089–94. doi: 10.1136/ard.2011.153718 21978999

[11] Romero R Kusanovic JP Chaiworapongsa T Hassan SS. Placental bed disorders in preterm labor, preterm PROM, spontaneous abortion and abruptio placentae. Best Pract Res Clin Obstet Gynaecol. (2011) 25:313–27. doi: 10.1016/j.bpobgyn.2011.02.006 21388889 PMC3092823

[12] American College of Obstetricians and Gynecologists. Gestational hypertension and preeclampsia: ACOG Practice Bulletin 222. Obstet Gynecol. (2020) 135:e237–60. doi: 10.1097/AOG.0000000000003891 32443079

[13] Lees CC Stampalija T Baschat A da Silva Costa F Ferrazzi E Figueras F . ISUOG Practice Guidelines: diagnosis and management of small-for-gestational-age fetus and fetal growth restriction. Ultrasound Obstet Gynecol. (2020) 56:298–312. doi: 10.1002/uog.22134 32738107

[14] American College of Obstetricians and Gynecologists. Management of preterm labor: Practice Bulletin 171. Obstet Gynecol. (2016) 128:e155–164. doi: 10.1097/AOG.0000000000001711 27661654

[15] Nagy-Vincze M Vencovsky J Lundberg IE Dankó K. Pregnancy outcome in idiopathic inflammatory myopathy patients in a multicenter study. J Rheumatol. (2014) 41:2492–4. doi: 10.3899/jrheum.140438 25452187

[16] Nagy-Vincze M Szinay D Szabó K Deliné SM Balkay L Béldi T . High fetal risk in pregnancies of myositis patients: a Hungarian cohort study. Front Lupus. (2024) 2:1449390. doi: 10.3389/flupu.2024.1449390

[17] Gutiérrez G Dagnino R Mintz G. Polymyositis/dermatomyositis and pregnancy. Arthritis Rheum. (1984) 27:291–4. doi: 10.1002/art.1780270307 6704192

[18] Silva CA Sultan SM Isenberg DA. Pregnancy outcome in adult-onset idiopathic inflammatory myopathy. Rheumatology. (2003) 42:1168–72. doi: 10.1093/rheumatology/keg318 12777640

[19] Kolstad KD Fiorentino D Li S Chakravarty EF Chung L. Pregnancy outcomes in adult patients with dermatomyositis and polymyositis. Semin Arthritis Rheum. (2018) 47:865–9. doi: 10.1016/j.semarthrit.2017.11.005 29217291 PMC5960607

[20] Tuccinardi A Czuzoj-Shulman N Abenhaim HA. Maternal and neonatal outcomes among pregnant women with inflammatory myopathies. J Perinat Med. (2022) 50:587–94. doi: 10.1515/jpm-2021-0361 35286050

[21] Che WI Hellgren K Stephansson O Lundberg IE Holmqvist M. Pregnancy outcomes in women with idiopathic inflammatory myopathy, before and after diagnosis-a population-based study. Rheumatology. (2020) 59:2572–80. doi: 10.1093/rheumatology/kez666 31998957 PMC7449806

[22] Doria A Iaccarino L Ghirardello A Briani C Zampieri S Tarricone E . Pregnancy in rare autoimmune rheumatic diseases: UCTD, MCTD, myositis, systemic vasculitis and Beçhet disease. Lupus. (2004) 13:690–5. doi: 10.1191/0961203304lu1098oa 15485105

[23] Akiyama C Shirai T Sato H Fujii H Ishii T Harigae H. Association of various myositis-specific autoantibodies with dermatomyositis and polymyositis triggered by pregnancy. Rheumatol Int. (2022) 42:1271–80. doi: 10.1007/s00296-021-04851-1 33837447

[24] Campanilho-Marques R Fonseca JE Machado PM. Treatment of idiopathic inflammatory myopathies. Joint Bone Spine. (2025) 92:105932. doi: 10.1016/j.jbspin.2025.105932 40545027

[25] Yang H Chen Q Sun C Jin Q Zhang L Liu Q . Clinical and prognostic associations of anti-Jo-1 antibody levels in patients with antisynthetase syndrome. Respir Res. (2024) 25:222. doi: 10.1186/s12931-024-02851-w 38811943 PMC11137886

[26] Tang K Zhou J Lan Y Zhang H Jin H. Pregnancy in adult-onset dermatomyositis/polymyositis: A systematic review. Am J Reprod Immunol. (2022) 88:e13603. doi: 10.1111/aji.13603 35867856

[27] Sammaritano LR Bermas BL Chakravarty EE Chambers C Clowse MEB Lockshin MD . 2020 American College of Rheumatology Guideline for the Management of Reproductive Health in Rheumatic and Musculoskeletal Diseases. Arthritis Rheumatol. (2020) 72:529–56. doi: 10.1002/art.41191 32090480

[28] ACOG Committee Opinion No. 743 summary: low-dose aspirin use during pregnancy. Obstet Gynecol. (2018) 132:254–56. doi: 10.1097/AOG.0000000000002709 29939936

[29] Tektonidou MG Andreoli L Limper M Amoura Z Cervera R Costedoat-Chalumeau N . EULAR recommendations for the management of antiphospholipid syndrome in adults. Ann Rheum Dis. (2019) 78:1296–304. doi: 10.1136/annrheumdis-2019-215213 31092409 PMC11034817

[30] Hamulyák EN Scheres LJJ Goddijn M Middeldorp S. Antithrombotic therapy to prevent recurrent pregnancy loss in antiphospholipid syndrome-What is the evidence? J Thromb Haemost. (2021) 19:1174–85. doi: 10.1111/jth.15290 33687789 PMC8252114

