# Supplementary material for: Pregnancy outcomes in idiopathic inflammatory myopathies: a Portuguese multicentre study
Source: Front Med (Lausanne). 2025 Dec 19;12:1724170. doi: 10.3389/fmed.2025.1724170 (PMC12758025; doi:10.3389/fmed.2025.1724170)
Supplement: Supplementary file 1 [file Table_1.docx]

**Supplementary Table S1.** Detailed baseline characteristics of all pregnancies at conception

| Pregnancy | IIM Subtype | Preconception treatment | PDN Dose (mg/day) | Disease duration, years | Disease activity at conception | CK at conception (U/L) | Skin DAS  (0-5) | MMT8  (0-150) |
| --- | --- | --- | --- | --- | --- | --- | --- | --- |
| 1 | Overlap (SSc–PM) | AZA, HCQ, PDN | 5 | 5 | Remission | <150 | 0 | 150 |
| 2 | Overlap (SSc–PM) | AZA, HCQ, PDN | 2.5 | 6 | Remission | <150 | 0 | 150 |
| 3 | Overlap (RA–PM) | HCQ, PDN | 15 | 22 | Remission | <150 | 0 | N/A |
| 4 | Overlap (PM–JIA) | AZA, PDN | 5 | 12 | Remission | <150 | 0 | 150 |
| 5 | ASyS | MMF, CYC, PDN (unplanned) | 20 | 7 | Active | 495 | 0 | 144 |
| 6 | ASyS | RTX, HCQ, cyclosporine, PDN | 10 | 3 | Active | <150 | 1  (periungual erythema) | 150 |
| 7 | ASyS | Tocilizumab, HCQ, cyclosporine, PDN | 7.5 | 4 | Active | <150 | 1  (Gottron’s papules) | 150 |
| 8 | DM | **Diagnosed during pregnancy** | | | | | | |
| 9 | DM | Chloroquine phosphate, PDN | 5 | 2 | Remission | <150 | 2  (periungual erythema, heliotrope rash) | 150 |
| 10 | DM | HCQ, AZA, PDN | 5 | 5 | Remission | <150 | 0 | 150 |
| 11 | IMNM | **Diagnosed during pregnancy** | | | | | | |
| 12 | PM | AZA, PDN | 5 | 5 | Remission | <150 | 0 | 150 |

**Abbreviations:** IIM, idiopathic inflammatory myopathies; ASyS, antisynthetase syndrome; DM, dermatomyositis; IMNM, immune-mediated necrotising myopathy; PM, polymyositis; SSc, systemic sclerosis; RA, rheumatoid arthritis; JIA, juvenile idiopathic arthritis; AZA, azathioprine; HCQ, hydroxychloroquine; PDN, prednisolone; MMF, mycophenolate mofetil; CYC, cyclophosphamide; RTX, rituximab; CK, creatine kinase; DAS, Disease Activity Score (skin); MMT8, Manual Muscle Testing-8.
