# Supplementary material for: Pregnancy outcomes in idiopathic inflammatory myopathies: a Portuguese multicentre study
Source: Front Med (Lausanne). 2025 Dec 19;12:1724170. doi: 10.3389/fmed.2025.1724170 (PMC12758025; doi:10.3389/fmed.2025.1724170)
Supplement: Supplementary file 2 [file Table_2.docx]

**Supplementary Table S2.** Detailed disease course, therapeutic management and pregnancy evolution

| Pregnancy | Treatment during pregnancy | Cumulative GC dose, mg | Flare during pregnancy | Peak CK during pregnancy (U/L) | Skin DAS  (0-5) | MMT8  (0-150) | Postpartum flare | Gestational age, weeks | Delivery mode | Birth weight, grams |
| --- | --- | --- | --- | --- | --- | --- | --- | --- | --- | --- |
| 1 | AZA, HCQ, GC, ASA | 1320.0 | None | <150 | 0 | 150 | None | 37.7 | Vaginal | 2520.0 |
| 2 | AZT, HCQ, PDN, ASA | 227.5 | CK elevation, myalgia, proximal weakness) | 1049 | 0 | 128 | Miscarriage at 6 weeks | | | |
| 3 | HCQ, PDN | 2230.0 | No | 732 | 0 | N/A | None | 39.0 | C-section | 3915.0 |
| 4 | HCQ, AZA, PDN | 350.0 | Periorbital erythema | <150 | 1 | 150 | N/A | Miscarriage at 10 weeks | | |
| 5 | MMF, CYC  (last infusion 2 weeks before conception), PDN | 840.0 | CK elevation | 434 | 0 | 144 | Miscarriage at 6 weeks | | | |
| 6 | Rituximab (3 weeks pre-conception), HCQ, cyclosporine, PDN, ASA | 2186.3 | Arthritis, mechanic’s hands | <150 | 1 | 150 | Arthritis | Stillbirth at 37.1 weeks | | |
| 7 | Tocilizumab, HCQ, cyclosporine, PDN, ASA, LMWH | 1770.0 | Arthritis, mechanic’s hands | <150 | 1 | 150 | None | 33.7 | C-section | 1745.0 |
| 8 | IVIG, AZA, GC, ASA | 10760.0 | Diagnosed during pregnancy | 1731 | 3 | 95 | None | 38.3 | C-section | 2900.0 |
| 9 | Chloroquine phosphate, PDN | 1365.0 | No | <150 | 2 | 150 | None | 39.0 | C-section | 2920.0 |
| 10 | HCQ, AZA, PDN | 280.0 | CK elevation | 587 | 0 | 150 | Miscarriage at 8 weeks | | | |
| 11 | PDN, AZA | 1030.0 | CK elevation, proximal weakness | 759 | 0 | 142 | Myalgia, proximal weakness | 40.3 | C-section | 3925.0 |
| 12 | AZA, PDN, ASA | 1365.0 | None | <150 | 0 | 150 | None | 39.0 | Vaginal | 3060.0 |

**Abbreviations**: AZA, azathioprine; HCQ, hydroxychloroquine; GC, glucocorticoids; ASA, acetylsalicylic acid (low-dose aspirin); PDN, prednisolone; MMF, mycophenolate mofetil; CYC, cyclophosphamide; RTX, rituximab; LMWH, low-molecular-weight heparin; IVIG, intravenous immunoglobulin; CK, creatine kinase; DAS, Disease Activity Score (skin); MMT8, Manual Muscle Testing-8; N/A, not available; APO, adverse pregnancy outcome; FGR, fetal growth restriction; SGA, small-for-gestational-age; C-section, caesarean section.
